# Experiences of families living with tuberculosis patients in the North West province, South Africa

**DOI:** 10.4102/hsag.v29i0.2530

**Published:** 2024-05-31

**Authors:** Keni J. Sebothoma, Mampheko D. Peu, Mmamphamo M. Moagi, Nombeko Mshunqane

**Affiliations:** 1 Department of Nursing, Faculty of Health Sciences, University of Pretoria, Pretoria, South Africa; 2 Department of Nursing Sciences, Faculty of Health Sciences, University of Limpopo, Sovenga, South Africa; 3 Department of Physiotherapy, Faculty of Health Sciences, University of Pretoria, Pretoria, South Africa

**Keywords:** families, health promotion, intervention, tuberculosis, disease, health

## Abstract

**Background:**

The families living with tuberculosis (TB) patients play a vital role in the care of these patients. Little is known about the experiences of families living with family members who are infected with TB.

**Aim:**

The aim of the study was to explore and describe the experiences of families having a member or members diagnosed with TB.

**Setting:**

The study was conducted in the Ngaka Modiri Molema district in the North West province of South Africa.

**Methods:**

This was a qualitative study using a descriptive phenomenological approach. Ten families with member(s) who had TB were purposively selected. Data were collected through face-to-face, semi-structured individual interviews that were recorded. Data were analysed using Colaizzi’s seven steps.

**Results:**

The following essential meanings emerged: family members’ caregiving experiences, family members’ challenging experiences, and family members’ health literacy experiences.

**Conclusion:**

Families had a lack of TB knowledge, which was associated with their poverty and with community health nurses not being committed to patient education. In poor, rural settings, nurses need to support families with adequate TB knowledge to limit the spread of TB and achieve the best treatment outcomes.

**Contribution:**

Family involvement is vitally important in TB health promotion. Health promotion is a crucial tool for achieving comprehensive health and social growth. Wider interventions concentrating on families are beneficial for promoting health and preventing TB.

## Introduction

Tuberculosis (TB) remains a global health concern as it continues to infect and claim the lives of people (Black, Amien & Shea 2018; Izudi et al. 2019). In 2019, the World Health Organization (WHO) estimated that 10.4 million and 558 000 people globally contracted TB and drug-resistant TB, respectively (Lwin, Apidechkul & Saising 2022). Eradicating TB has become a global health priority; however, the WHO continues to report 10 million new cases annually (Tadokera et al. 2020). According to Boru, Shimels and Bilal (2017), TB is the second leading cause of death from among infectious and communicable diseases following human immunodeficiency virus (HIV). Tuberculosis is a disease that affects the poor, who are socially and economically disadvantaged (Datiko et al. 2019).

South Africa (SA) is a middle-income country and is characterised by a low life expectancy, which is reportedly 55.3 years for men and 60.4 years for women (Perez et al. 2013). South Africa reports the highest number of TB cases, globally, largely owing to having a large burden of people living with HIV (Makhado et al. 2018). The WHO reported a decrease in the global trend of TB; however SA continues to have an intolerably high incidence of active TB infection (Kigozi et al. 2017).

Human immune deficiency virus is a primary driver of TB infection and in some African countries, 70% of people living with TB correspondingly have an HIV co-infection (Yoko et al. 2017). The persistent nature of TB makes the infection difficult to treat, which results in increased morbidity and mortality, as well as the emergence of treatment-resistant TB (Venter 2018). Approximately 18% of TB cases are unsuccessfully treated, which may lead to the development of multi-drug resistant TB (MDR-TB) (Wademan et al. 2021). South Africa has a significant burden of drug-resistant TB. In 2015, 20 000 people were reportedly diagnosed with rifampicin-resistant TB (Cox et al. 2017). The development of treatment-resistant TB can be mitigated through timely detection, diagnosis, and treatment.

In SA, several provinces have experienced a delay in starting treatment (Cox et al. 2017). The gap in finding and managing TB infection continues (WHO 2018). Several low- and middle-income countries experience late diagnosis and management of TB, leading to unsuccessful management and defaulted treatment (Li et al. 2014). To control TB successfully, families play a vital role in supporting and caring for TB patients (Kristinawati, Muryadewi & Irianti 2019). Caregivers’ attitudes and knowledge play a significant role in TB transmission, which is driven by social and environmental factors.

In the North West province of SA, TB is a leading cause of death, resulting in 9% of deaths, against a target of 5% outlined in the Directly Observed Treatment Strategy (DOTS) (North West Department of Health 2022). In 1991, the WHO introduced the DOTS strategy globally, which proved to be cost-effective in averting TB infections and deaths (Naidoo & Mwaba 2010). The DOTS strategy has not been as successful in the North West province, presumably owing to a lack of communication between nurses and patients (Serapelwane, Davhana-Maselesele & Masilo 2016). It was further reported that the health education was of low quality, as patients received their medication without the necessary information on how to take it, which may have resulted in reduced TB cure rates and frequent re-hospitalisations (Serapelwane et al. 2016). Although facility-based diagnosis and treatment commencement of TB has improved in the North West province, the control of TB transmission in the community remains poor.

Given the pressing challenges of managing TB effectively in the North West province, the researcher explored and described the experiences of families living with a member, or members, diagnosed with TB. This was a qualitative, descriptive phenomenological study.

## Context of the study

This study was conducted in the villages and townships of the Ngaka Modiri Molema district. The district consists of five sub-districts, namely Mafikeng, Ditsobotla, Ramotshere Moiloa, Ratlou, and Tswaing. The study was conducted in the towns and villages of Ngaka Modiri Molema district because of the high burden of TB.

## Research methods and design

### Population and sampling

The population for this study comprised families having a member or members diagnosed with TB in the Ngaka Modiri Molema district. The participants comprised 10 family members from the Ngaka Modiri Molema district, with two family participants from each sub-district.

### Inclusion criteria

The family member had to be 18 years and older and must have had a family member suffering from TB for more than 5 months.

### Exclusion criteria

Family members younger than 18 years and not staying with a TB patient at the time of the study were excluded.

Participants were purposively sampled. Participation was voluntarily, and participants signed informed consent forms. The participants were recruited from the clinics and community health centres when they accompanied TB patients for follow-ups. A date and time were set for interviews with participants who were willing to participate. During the data collection, the researcher engaged in reflexivity in order to comprehend and articulate positionality. The researcher maintained a reflective attitude towards the whole research process by questioning his prior understanding of data and themes (preconceptions). The researcher compared the original data with the derived text of themes. Initially the researcher adopted the position of an outsider and later an insider during follow up visits. The researcher further bracketed his previous beliefs, values, and knowledge, remained neutral in order to eliminate affecting or influencing the research and listened to the real voices of the participants.

### Data collection

The researcher conducted in-depth, semi-structured, individual, face-to-face interviews with family members using an interview guide. The researcher posed the following questions to the family members:

‘What are the experiences of families having a member(s) diagnosed with TB in the North West province?’

The researcher asked the participants to describe their TB-related experiences, their interactions with community health nurses (CHNs), and clinics or community health centres. The question was developed in English and translated to the Tswana language. The researcher followed these questions with probing questions. The researcher used multiple interview skills such as probing, listening, resaying, and clearing up (Gray, Grove & Sutherland 2017). Data were collected from 30 min to an hour until saturation was achieved and the interview was audio-recorded for accuracy.

Audio recording, observation, and documenting field notes supported data collection. The researcher collected data at the participants’ homes as they opted to be interviewed at their homes. This assisted the researcher to observe and understand the day-to-day lives of the participants in their real and natural settings.

### Pilot study

Before collecting data, the researcher conducted a pilot study with two family members. The two participants followed the process of informed consent and confidentiality. Participants in the pilot study did not participate in the main study to eliminate bias as their experience in the pilot study may result in bias in the research in the main study. The questions were pretested to identify pitfalls so that they could be refined if the need arose.

### Data analysis

The Colaizzi (1978) method of data analysis was followed to reveal the deeper meanings of the participants’ experiences (Polit & Beck 2017). The interviews were transcribed verbatim and all printed transcriptions were read repeatedly to grasp their essential meanings, and relevant statements were extracted. The researcher also expressed important statements and classified formulated meanings into themes and/or patterns and sub-themes. The results were combined into a comprehensive description of the phenomenon. The final analysis was returned to the participants to affirm the results (Polit & Beck 2017, 2018).

### Measures of Trustworthiness

Trustworthiness was achieved by using the criteria of credibility, dependability, transferability, and confirmability (Polit & Beck 2018). Credibility was achieved through long engagement with the participants. The researcher maintained confidentiality of the participants by using pseudonyms (e.g., family 1 [FM1]). The researcher used audiotape to collect data which were kept under lock and key all the time and were accessible to the researcher only. The results of the interviews were reported and published with the permission of the participants. The interviews lasted from 30 min to an hour until saturation was achieved. After transcription, the researcher asked the participants to check the transcriptions (member checking). The researcher achieved member checking by taking back the results of the interviews to the family participants so that the participants can confirm the interpretations and for adequacy. The researcher further deliberated data-collection issues with peers (peer debriefing). The researcher collected data by using various means and sources (data triangulation) for example semi-structured interviews, observations, and field notes. Dependability was enhanced by recording and transcribing the data verbatim. There was consensus between the supervisor and researcher about the results and their findings. Confirmability was affirmed with audio-taped data, field notes, and interview report. Transferability was achieved through purposive sampling of participants.

### Ethical considerations

This study was approved by the Research Ethics Committee for clearance (Ethical reference no. 480/2020). Permission to conduct the study was obtained from the Provincial Department of Health, and the management of the healthcare facilities. All participants signed an informed consent form before participation. Furthermore, the researcher ensured that ethical issues including justice, respect for human dignity, and beneficence were observed during the study. Justice was observed by treating all the participants equally and privacy was maintained by using pseudo names such as FM1 and interview recordings were kept under lock and key without access to others. Respect for human dignity was observed by providing participants with adequate information and allowing them to make informed decisions. Beneficence was observed by avoiding physical and emotional harm during data collection.

## Results

### Participants’ demographic data

The researcher interviewed 10 participants from the Ngaka Modiri Molema. Most of the participants (80%) were unemployed and from the villages (Table 1). The themes and sub-themes that emerged from the interviews are displayed in Table 2.

**TABLE 1:** Participants’ demographic data.

Participant number	Age (years)	Family composite	Employment	House or environment
Family 1	55	Extended family	Unemployed	Village
Family 2	64	Extended family	Pensioner	Village
Family 3	50	Nuclear family	Unemployed	Village
Family 4	31	Nuclear family	Unemployed	Township
Family 5	83	Extended family	Pensioner	Township
Family 6	47	Extended family	Unemployed	Village
Family 7	40	Extended family	Unemployed	Village
Family 8	35	Extended family	Hairdresser	Village
Family 9	45	Extended family	Unemployed	Village
Family 10	56	Nuclear family	Self employed	Village

**TABLE 2:** Essential meanings that emerged from participants experiences of having a member(s) diagnosed with tuberculosis.

Themes	Sub-themes
1: Family members’ caregiving experiences	1.1 Physical care and support of the patient 1.2 Personal TB protection 1.3 TB treatment adherence
2: Family members’ challenging experiences	2.1 Financial challenges 2.2 Healthcare access challenges
3: Family members’ TB health literacy experiences	3.1 Community health nurses’ ineffective health education 3.2 Sources of TB information

TB, tuberculosis.

#### Theme 1: Family members’ caregiving experiences

Family members play a central role in TB care as they support patients who are receiving a long course of TB treatment. Family care and support can prevent patients from defaulting on treatment. This theme had three sub-themes: physical care and support of the patient, experiences of personal TB protection, and experiences of TB treatment adherence.

**Sub-theme 1.1: Physical care and support of the patient:** Participants described their lived experiences of assisting family members with the preparation of food, administration of medication, treatment adherence, personal and environmental cleanliness, as well as divine support. The responses of participants FM03 and FM05 were in agreement: ‘I assist my husband by ensuring that he is taking his medication on time and that he is eating well. I also discourage him from smoking as he is a smoker. I further encourage him to go to the clinic to fetch his medication’. Other individual responses included:

‘When I wake up in the morning, I open the windows. I gave him food and his medication. I then prepare water for him to bathe and take him out of the room as he cannot walk. I support him with the arm and put him in the sitting room.’ (FM01, 55 years old, Extended family, Unemployed, Village)

‘We wash his clothes; we cook the right food for him and make sure that he eats on time and takes his medication.’ (FM02, 64 years old, Extended family, Pensioner, Village)

‘I was always praying for her. My support for her was through prayer, and I was really praying.’ (FM04, 31 years old, Nuclear family, Unemployed, Township)

**Sub-theme 1.2: Experiences of personal tuberculosis protection:** The participants indicated that they are mindful of the fact that TB can spread to other family members, mainly children. The participants further noticed that other family members must know how to shield themselves from contracting TB. Furthermore, participants confirmed that good coughing etiquette and the wearing of masks are essential to stop the spread of TB. Most participants in this study described a knowledge deficit concerning personal TB protection, as described by participants FM08 and FM04: ‘They said the other kids are not under five, so I just thought that the kids could not be infected. We did not protect ourselves, and it went well just fine. I just gave her food and washed her clothes and there were no problems. We do not protect ourselves’.

Participants FM07 and FM01 reported how they protected themselves from contracting TB: ‘We protect ourselves by wearing masks. He is eating his food and is not sharing with the kids or leaving his food to be eaten by the kids. He has his cutlery and does not share it with anybody’. Participant FM02 related the following:

‘He sleeps alone in a separate room. We open the windows for fresh air during the day. We make sure that he spends some time outdoors. He covers his nose and mouth with a tissue when sneezing, coughing, or laughing. He covers the tissues with plastic and throws them in a dustbin. We even cough in our elbows when we are not having the tissue and wash our hands with water and soap. He does not cough in his hands at all.’ (FM02, 64 years old, Extended family, Pensioner, Village)

**Sub-theme 1.3: Experiences of tuberculosis treatment adherence:** Taking the recommended medicine for the recommended time is essential for avoiding drug resistance and recuperating from TB. The participants described their roles of ensuring treatment adherence by reminding them, supervising them, and cooking food for them before taking medication, as depicted by participants FM01, FM02 FM03, FM05 and FM08: ‘When I wake up in the morning I give him food, give him his medication, and talk to him to make sure that he swallowed the treatment. I also provide him with words of encouragement to take the pills regularly for six months even when he feels better’. Participant FM09 recounted:

‘We encourage him to take his treatment, but because they are big he wants to vomit them; thus, we break them for him and ask him to take them with a lot of water.’ (FM09, 45 years old, Extended family, Unemployed, Village)

#### Theme 2: Family members’ challenging experiences

Tuberculosis results in many problems for families. Patients need to eat a proper diet to recuperate. Furthermore, patients need to go for regular check-ups at health facilities to fetch medication and monitor their weight and blood pressure. Participants highlighted the fact that patients could not continue to work while suffering from TB. Thus families have to work and look after their relatives who have been diagnosed with TB. Family members’ challenging experiences emerged as the second theme, with two sub-themes: family members’ financial challenges and healthcare access challenges.

**Sub-theme 2.1: Financial challenges:** The participants described their lived experiences of financial suffering and a lack of access to social grants as challenges, as patients cannot work while suffering from TB. Other participants voiced their view that the grants are not sufficient to take care of their necessities. The responses of the participants in this regard are as follows:

‘He just received his first grant recently. There is no relief yet as he just obtained it recently and there was a huge gap, and I don’t see any difference yet. I will see as time goes by, how to re-arrange things.’ (FM01, 55 years old, Extended family, Unemployed, Village)

‘When she goes to the clinic to fetch treatment, I hire transport for her. In this house, it’s my husband and me who are getting old age pension; She went to fetch the forms [*patient*], and she was told that they no longer provide a social grant for TB as it is curable. On my social grant, there are two kids I adopted … with our grants … Food is expensive, and electricity is worse. My husband has a chronic disease, and every month we must send a child to fetch treatment for him at the hospital. It is money.’ (FM05, 83 years old, Extended family, Pensioner, Village)

‘The challenges are there, we do get social grants for the kids, but they are not enough. I have three kids going to school, and their dad is not working. The grant is not enough at all. There is always a shortage of food.’ (FM08, 35 years old, Extended family, Hairdresser, Village)

**Sub-theme 2.2: Healthcare access challenges:** Participants described their experiences with transport. Most of the participants could not afford public transport as they were unemployed, thus, accessing healthcare facilities is a problem. Participants FM01, FM03, FM05 and FM06 supported the findings by collectively stating: ‘The challenge I have with TB is that I am staying alone with him. I am the only one taking care of him. I have three kids. I am the one fetching treatment for him from the clinic. I do not have the income to get transport to take him to the clinic. At times they want me to take him to the clinic to check his blood pressure and weight. I do have a transport challenge. I do not have an income, as I do not work and depend on the children’s grants’. An additional response from participant FM05 follows:

‘I am eighty-three years old, and I use crutches to walk. I am also a patient. I must also hire transport for her to the clinic for TB check-ups. My husband also has a chronic disease, and every month we send a child to fetch treatment for him with transport. I have a back problem too, and I also fetch my treatment from a different hospital. On top of that, I fetch treatment for hypertension from the clinic. Every month we spend money to fetch treatments and for her to do check-ups for TB.’ (FM05, 83 years old, Extended family, Pensioner, Township)

#### Theme 3: Family members’ tuberculosis health literacy experiences

Tuberculosis health literacy is vital for attaining knowledge and skills required by families to enable them to obtain, understand, and apply TB health knowledge. Being knowledgeable will assist families in promoting and preventing the spread of TB. Family members need to know the signs and symptoms of TB. Family members’ TB health literacy experiences emerged as the final theme with two sub-themes: Family members’ experiences of CHNs’ ineffective health education and experiences of sources of TB information.

**Sub-theme 3.1: Experiences of community health nurses’ ineffective health education:** Participants described their lived experiences of CHNs’ failure to inform them about TB. Most participants stressed that CHNs supply them with medication without any information. Participants FM07, FM09 and FM10 confirmed the findings by stating: ‘They only give us treatment. They did not explain to me. I thought it was still meningitis. He [*my son*] also did not mention that he was having TB. They just want to push the queue and finish their work’. Participant FM04 stated:

‘They did not come to inform us about TB. He was just given treatment.’ (FM04, 31 years old, Nuclear family, Unemployed, Township)

**Sub-theme 3.2: Experiences of sources of tuberculosis information:** Most participants described their lived experiences of not being informed by CHNs concerning TB causes, signs and symptoms, treatment, and side-effects of treatment and how to prevent the transmission of TB. Responses from Participants FM03, FM04 and FM09 included: ‘They did not inform us about TB. He was just given treatment for TB. They only give us treatment’. One participant though had direct experience with TB by knowing someone who suffered from TB:

‘I know about TB as my aunt had it and she passed on. So, I know about TB from home.’ (FM01, 55 years old, Extended family, Unemployed, Village)

## Discussion

The study revealed that family members play a vital role in caring for TB patients and preventing the spread of TB in communities. The discussion focuses on the following themes: Family members’ caregiving experiences, family members’ challenging experiences, and family members’ TB health literacy experiences and their sub-themes

### Family members’ caregiving experiences

This theme was divided into three sub-themes: family members’ physical care and support of the client, family members’ experiences of personal TB protection, and family members’ experiences of TB treatment adherence.

### Family members’ physical care and support of the client

In this study, the families assisted TB patients with personal and environmental hygiene to ensure an efficient recovery. They ensured that the patients ate nutritive meals before treatment to speed up the healing process and to live healthily. Fana and Sotana (2021) highlighted the importance of family care to the TB patient during their recovery. Therefore, family units should be knowledgeable regarding TB to enable the best possible care for the patient. The families supported patients with TB physically, emotionally, and spiritually to ensure that they adhere to TB treatment to reduce the transmission of TB. Kristinawati et al. (2019) and Gyimah and Dako-Gyeke (2019) added that the family should foster a culture of empathy, deference, and reassurance to encourage adherence with the TB regimen. Vanleeuw, Zimbe-Mkalibe and Atkins (2022) and Herdianti, Entianopa and Surgiarto (2020), emphasised the importance of good family support in terms of daily household tasks and motivation to prevent defaulting on treatment and help in improving treatment outcomes.

### Family members’ experiences of personal tuberculosis protection

Participants demonstrated knowledge deficit with regard to personal TB protection. The level of education is associated with the knowledge of TB as most participants in the study had a lesser level of education. Herdianti et al. (2020) emphasised that the family should be knowledgeable in TB prevention in order to take precautionary measures for themselves and their environment. Bedingfield et al. (2022), in addition stated that the gaps in TB education and counselling have a negative impact on the patients and their families as they are meant to correct misconceptions about TB and reduce the transmission. In addition, Alberta and Widyastuti (2021), Sulistyono, Susanto and Tristiana (2020), found that personal TB protection was not practiced in Indonesia.

### Family members’ experiences of tuberculosis treatment adherence

In this study, participants stressed the need for patients to adhere to treatment in order to improve their TB treatment outcome. There were few community health workers to supervise TB patients at home. However, the families ensured that their members adhere to treatment by reminding them to take their medication, monitored them, and prepared food so that they do not take treatment before having meals. Ukwaja et al. (2017), found that some patients had family support and the others had no support at all. Sahile, Yared and Kaba (2018); Alberta and Widyastuti (2021); Nastiti and Kurniawan (2020), with findings similar to this study, mentioned the relation between family support and TB treatment adherence.

### Family members’ challenging experiences

In this study, the family and the TB patients were faced with challenges that may have a negative impact on the TB treatment outcome. The challenges were subdivided into family members’ financial and social grant challenges and family members’ healthcare access challenge.

#### Family members’ financial and social grant challenges

In this study, 80% of the families did not have an income and experienced financial constraints. They received financial support in the form of social grants, but this was not enough to meet their daily needs for the whole month. In addition, Vanleeuw (2022) cited that only 5% of TB patients in SA receive disability grants. Fana and Sotana (2021) stressed that in order to improve TB outcome, the challenges faced by families caring for TB patients need to be addressed.

#### Family members’ healthcare access challenges

Most of the participants could not afford public transport because lack of income and accessing healthcare facilities was a challenge. This was echoed by Yellappa et al. (2016) who alluded that patients from villages are grappling to access healthcare facilities because of lengthy distances and financial constraints as compared to patients in cities. In contrast to these findings, Sahile et al. (2018) indicated that healthcare access was not a problem as patients and their families resided nearby.

### Family members’ tuberculosis health literacy experiences

Tuberculosis patients, their families, and the community need to be provided with adequate TB information in order to seek medical care in time. This theme was subdivided into family members’ experiences of CHNs’ ineffective health education and family members’ experiences of sources of TB information.

#### Family members’ experiences of community health nurses’ ineffective health education

Most participants indicated that community health nurses fail to inform them about TB. The participants expressed that nurses supply them with TB drugs without TB treatment or TB information. De Queiroz et al. (2016) mentioned that the families are not considered in healthcare services in support of the findings. Sulistyono et al. (2020) highlighted the fact that the families and the community lack TB-related information and recommended research to deal with ways to inform the people about TB. In contrast, the study conducted by Gyimah and Dako-Gyeke (2019), in Ghana found that the participants had good TB knowledge; however they still believed the cause was related to a spiritual enemy. In addition, the study conducted in Saudi Arabia by Alotaibi et al. (2019) cited that healthcare providers had no TB knowledge and had poor relationship with the patients and their families.

#### Family members’ experiences of sources of tuberculosis information

Participants expressed that CHNs are not informing them about TB with regard to causes, the spread, prevention, and treatment. In contrast, Yermi et al. (2018) found that the families had good TB knowledge, and alluded that the family’s TB knowledge is the key in TB prevention. Vanleeuw et al. (2022) indicated that nurses need to be empowered with in-service education in order to provide effective health education. Dobler et al. (2018) emphasised that healthcare providers need to undergo first-line TB treatment to prevent unnecessary abortions to pregnant women as pregnant women are unaware that TB treatment is safe.

### Limitations

The study had a small sample size, and it was conducted in one district of the North West province. The results cannot be generalised to other settings.

## Conclusion

In the North West province of SA, families with a member(s) diagnosed with TB remain uninformed about TB. The families experience anguish, immeasurable pain, sorrow, and suffering as most families are unemployed.

### Recommendations

Based on the results of this study, some recommendations are made. These include: National and provincial departments of health should offer opportunities for periodical training of healthcare providers to enhance the quality of TB counselling and health education. Tuberculosis managers should provide adequate personnel to make time for one-on-one counselling and education. The families and the community should be provided with skills to plant vegetables to eat fresh vegetables and live healthily. The families of TB patients should be assisted with weekly food parcels to supplement TB treatment.
